# Comparison of a machine and deep learning model for automated tumor annotation on digitized whole slide prostate cancer histology

**DOI:** 10.1371/journal.pone.0278084

**Published:** 2023-03-16

**Authors:** Savannah R. Duenweg, Michael Brehler, Samuel A. Bobholz, Allison K. Lowman, Aleksandra Winiarz, Fitzgerald Kyereme, Andrew Nencka, Kenneth A. Iczkowski, Peter S. LaViolette

**Affiliations:** 1 Department of Biophysics, Medical College of Wisconsin, Milwaukee, Wisconsin, United States of America; 2 Department of Radiology, Medical College of Wisconsin, Milwaukee, Wisconsin, United States of America; 3 Department of Pathology, Medical College of Wisconsin, Milwaukee, Wisconsin, United States of America; 4 Department of Biomedical Engineering, Medical College of Wisconsin, Milwaukee, Wisconsin, United States of America; Jordan University of Science and Technology, JORDAN

## Abstract

One in eight men will be affected by prostate cancer (PCa) in their lives. While the current clinical standard prognostic marker for PCa is the Gleason score, it is subject to inter-reviewer variability. This study compares two machine learning methods for discriminating between cancerous regions on digitized histology from 47 PCa patients. Whole-slide images were annotated by a GU fellowship-trained pathologist for each Gleason pattern. High-resolution tiles were extracted from annotated and unlabeled tissue. Patients were separated into a training set of 31 patients (Cohort A, n = 9345 tiles) and a testing cohort of 16 patients (Cohort B, n = 4375 tiles). Tiles from Cohort A were used to train a ResNet model, and glands from these tiles were segmented to calculate pathomic features to train a bagged ensemble model to discriminate tumors as (1) cancer and noncancer, (2) high- and low-grade cancer from noncancer, and (3) all Gleason patterns. The outputs of these models were compared to ground-truth pathologist annotations. The ensemble and ResNet models had overall accuracies of 89% and 88%, respectively, at predicting cancer from noncancer. The ResNet model was additionally able to differentiate Gleason patterns on data from Cohort B while the ensemble model was not. Our results suggest that quantitative pathomic features calculated from PCa histology can distinguish regions of cancer; however, texture features captured by deep learning frameworks better differentiate unique Gleason patterns.

## Introduction

Prostate cancer (PCa) is the most diagnosed non-cutaneous cancer in men, affecting an estimated 268,000 men in 2022 [1]. Improved prostate cancer screening and therapies have led to a high five-year survival rate and the overall prognosis for PCa is one of the best compared amongst all cancers. Prostate cancer is currently graded using the Gleason grading system, assigning scores corresponding to the two most predominant morphological patterns present. More recently, it has been used to assign patients into one of five Grade Groups (GG) to predict prognosis [2, 3]. Clinically significant cancer (GG ≥ 2, tumor volume ≥ 0.5 mL, or stage ≥ T3) is often treated with radiation therapy and/or radical prostatectomy [4, 5]. Low-grade cancer can often be monitored through annual prostate specific antigen (PSA) testing. Side effects from prostate cancer treatment can include long-term complications such as impotence and impaired urinary function [6], thus early and accurate detection of PCa is necessary to minimize overtreatment while still addressing clinically significant cancer.

Digital pathology is playing an increasingly important role in clinical research, with applications in diagnosis and treatment decision support [7]. Fast acquisition time, management of data, and interpretation of histology has made digital pathology popular and easier for pathologists to manage and share slides. Additionally, artificial intelligence (AI) with digital pathology has created opportunities to incorporate computational algorithms into pathology workflows or for AI-based computer-aided diagnostics [8]. Machine learning has shown potential in decision-support systems, and in prostate cancer, can be applied to diagnostic imaging, surgical interventions, and risk assessment [9].

In prostate cancer research, many machine learning applications have been focused on automated Gleason grading. While the Gleason score is currently the gold standard prognostic marker for prostate cancer, the process of assigning grades is a subjective, quantitative metric. Additionally, pathologist-provided annotations for digital pathology studies is not only time consuming, but can result in significant inter-observer variability [10, 11]. The primary focus of these automated Gleason grading methods has been on biopsies or tissue microarrays as opposed to whole-slide images [12–15]. A fast, automated tool for identifying Gleason patterns in prostate histology could allow for rapid annotation and grading, as well as provide important prognostic information such as recurrence probabilities.

Previous studies have used a myriad of machine and deep learning architectures for Gleason pattern annotations. Machine learning models are often chosen based on overarching considerations of the model including size of training data, accuracy or interpretability of the output, training time, linearity, and number of features [16]. Data sets with fewer observations and higher number of features may benefit from using algorithms with high bias and low variance such as linear regression, Naïve Bayes, and linear SVM, whereas KNN, decision trees, and kernel SVMs work well with large data sets. Linear regression models are highly interpretable but are restricted to generate only linear functions, compared to a KNN which considers all input data. Faster training time is achievable with models such a Naïve Bayes and linear and logistic regressions; SVM, neural networks, and decisions trees require lots of time to train the data but may see increased accuracy.

Deep supervised learning models, such as convolutional neural networks, are of the simpler deep learning techniques and can generate a data output with provided labels; however, the decision boundaries may be impacted by imbalanced data. AlexNet takes in an RGB image, augments data to reduce over-fitting, normalize neighborhood pixels, and using a drop-out layer to avoid over-fitting [17]. VGGNet reduces the number of variables used in AlexNet for faster learning and is more robust to over-fitting [18]. ResNet finds simpler mapping by employing Identity and Projection shortcuts which push learned maps through the network [19]. This framework has one of the highest accuracies compared to the others but requires more training time and energy.

In this study, we developed an Automated Tumor Assessment of pRostate cancer hIstology (ATARI) classification model for the Gleason grading of whole-mount prostate histology using quantitative histomorphometric features calculated from digitized prostate cancer slides. The results of this model were validated using ground truth pathologist annotations. In addition, we compared this model to a residual network with 101 layers (ResNet101) for automated Gleason grading [20]. Specifically, we tested the hypothesis that a machine learning model applied to second-order features calculated from digitized histology could discriminate prostate cancer from normal tissue. We also hypothesized that deep learning model would differ in classification accuracy, both in detecting cancer and differentiating Gleason patterns.

## Materials and methods

### Patient population and data acquisition

Data from 47 prospectively recruited patients (mean age 59 years) with pathologically confirmed prostate cancer were analyzed for this study. This study was conducted according to the guidelines of the Declaration of Helsinki and approved by the Institutional Review Board of the Medical College of Wisconsin. Written informed consent was obtained from all subjects involved in the study. The data presented in this study are available on request from the corresponding author. The data are not publicly available due to patient privacy concerns. For model development, subjects were split into 2/3 training (n = 31 patients) and 1/3 testing (n = 16 patients) data sets, matched for tumor grade and other clinical characteristics (see Table 1).

**Table 1:** Patient demographics.

	Training	Testing
	(n = 31)	(n = 16)
Age at RP, years (mean, SD)	59 (6.8)	59 (4.9)
Preoperative PSA, ng/mL (mean, SD)	7.9 (6.2)	7.7 (4.5)
Grade group at RP (n, %) (n = 72)		
6	8 (26)	2 (12)
3+4	13 (41)	7 (44)
4+3	4 (13)	3 (19)
8	3 (10)	1 (6)
≥ 9	3 (10)	3 (19)

Clinicopathological features of the study cohort at the time of radical prostatectomy (RP).

### Tissue collection and processing

Prostatectomy was performed using a da Vinci robotic system (Intuitive Surgical, Sunnyvale, CA) [21, 22]. Whole prostate samples were fixed in formalin overnight and sectioned using custom axially oriented slicing jigs [23]. Briefly, prostate masks were manually segmented from the patient’s pre-surgical T2-weighted magnetic resonance image using AFNI (v.19.1.00) (Analysis of Functional NeuroImages, http://afni.nimh.nih.gov/) [24]. Patient-specific slicing jigs were modeled using Blender 2.79b (https://www.blender.org/) to match the orientation and slice thickness of each patient’s T2-weighted image [10, 25–27], and 3D printed using a fifth-generation Makerbot (Makerbot Industries, Brooklyn, NY). The MRI scans were not used beyond slicing molds for the remainder of this study.

**Fig 1:**
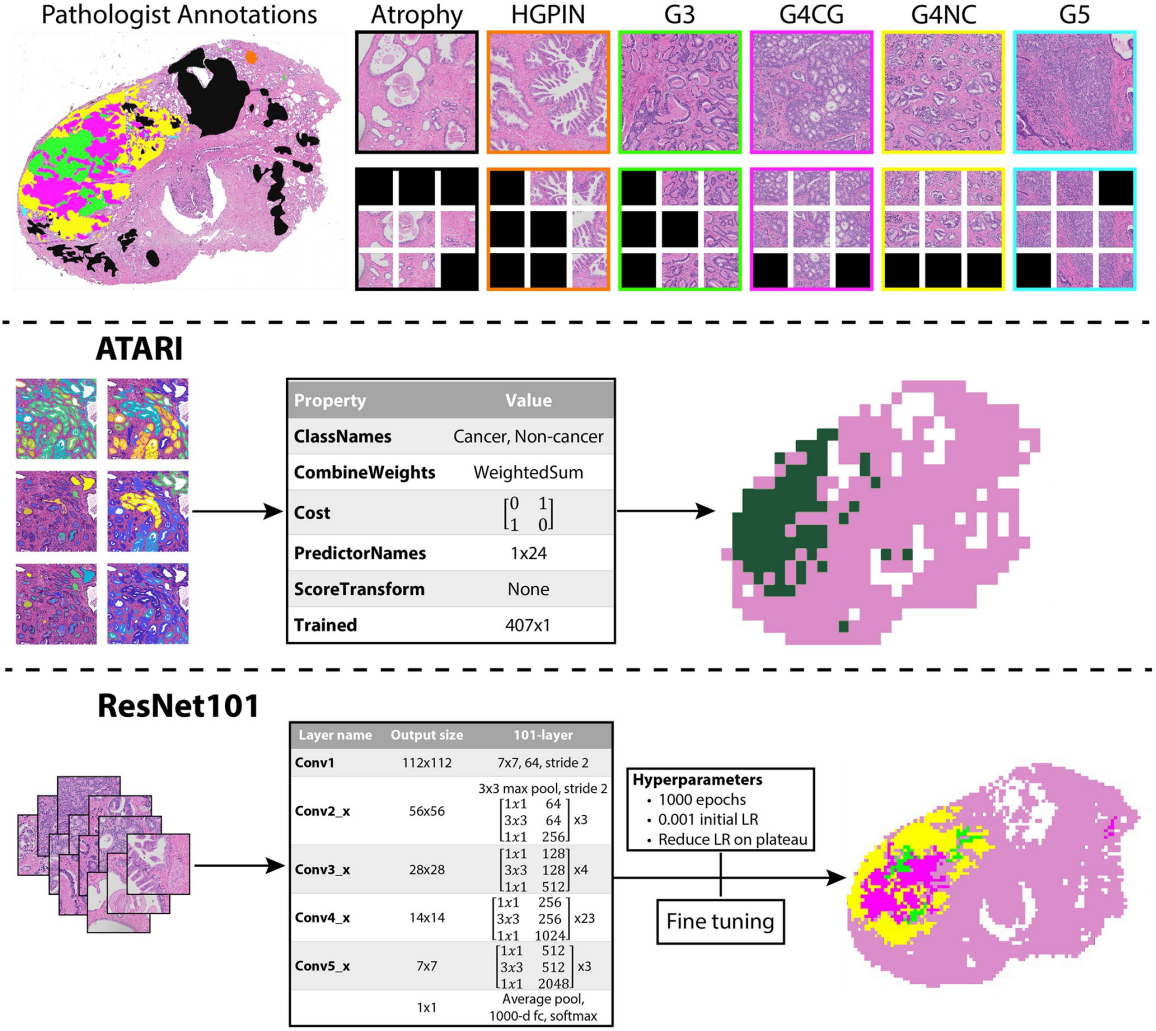
Model designs. Top: Annotation and tile extraction process. After manual annotation of digitized slides, 3000x3000 pixel tiles are extracted from unique annotated regions. Those tiles are then further divided into 1024x1024 pixel tiles and those that remain within a mask are saved (black tiles indicate unsaved tiles). Middle: Workflow for the ATARI classifier. Quantitative pathomic features calculated from the large tiles are used as input to a compact classification ensemble to predict cancer vs non-cancer in a whole-slide image. Bottom: Workflow for the ResNet101 classifier. 1024x1024 pixel annotated tiles are used as input into the ResNet model to predict non-cancer vs Gleason grade groups. Abbreviations: HGPIN = high-grade prostatic intraepithelial neoplasia; G3 = Gleason pattern 3; G4CG = Gleason pattern 4 cribriform; G4NC = Gleason pattern 4 non-cribriform; G5 = Gleason pattern 5.

Whole-mount tissue sections were processed, paraffin embedded, and resulting whole mount slides were hematoxylin and eosin (H&E) stained. The slides were then digitally scanned using a slide scanner (Olympus America Inc., Center Valley, PA, USA) at a resolution of 0.34 microns per pixel (40x magnification) to produce whole slide images (WSI), and down-sampled by a factor of 8 to decrease processing time. A total of 330 digitized slides were manually annotated using a Microsoft Surface Pro 4 (Microsoft, Seattle, WA, USA) with a pre-loaded color palette for different Gleason patterns [2] by a GU fellowship-trained pathologist (KAI). An example of the prostate annotation process is shown in Fig 1.

### Annotation segmentation

Digital whole-mount slides were divided into high resolution tiles that were 3000x3000 pixels and labeled using their corresponding xy-coordinates within the image. This size tile was chosen as it is the smallest resolution that our pathologist could determine Gleason grades. These tiles were then stitched back together to recreate the whole-mount image while concurrently creating x- and y-coordinate look-up tables. A subset of slides was rescanned on the Olympus slide scanner, and annotations that were performed on lower resolution digitized versions of the slide were quantitatively transferred (n = 201 slides). Briefly, the analogous annotated image was aligned to the newly digitized slide using MATLAB 2021b’s *imregister* function (The MathWorks Inc., Natick, MA, USA). The annotations were isolated to create a single mask for each of eight possible classes: seminal vesicles, atrophy, high-grade prostatic intraepithelial neoplasia (HGPIN), Gleason 3 (G3), Gleason 4 cribriform gland (G4CG), Gleason 4 non-cribriform glands (G4NC), Gleason 5 (G5), and unlabeled benign tissue. Gleason 4 patterns have been separated in our annotations as there are notable prognostic differences between the cribriform and non-cribriform patterns [28–31]. An additional averaged white image of non-tissue (i.e., background, lumen, and other artifacts) was found to remove these areas from the annotation masks to ensure the most representative histology remained for analysis. Each region of interest (ROI) within an individual class was individually compared to the xy-look-up tables to determine coordinates corresponding to tiles, and only those with over 50% of a specific annotation were included. Five tiles from each ROI were saved into annotation-specific directories for use with the ATARI model, except for unlabeled benign tissue where 15 tiles were randomly saved from each slide. ROIs that were too small to extract 5 tiles from were excluded.

Each annotated tile was further divided into 1024x1024 pixel tiles at the same image resolution for use with the ResNet101 model, resulting in upwards of 9 sub-tiles used for the ResNet101 per full-sized tile used for the ATARI model. Sub-tiles that remained within a mask were saved into annotation-specific directories, similarly to the large tiles used for the ATARI model. The ResNet101 additionally was trained using background tiles determined by areas that were included in the average white image. Tiles used for training were augmented by resizing (250x250 pixel), random cropping (240x240), applying color jitter (0.3, 0.3, 0.3), adding random rotations (±0-30º), applying random horizontal and vertical flips and center cropping to the ResNet input size of 224x224 as well as normalizing to ImageNet’s mean (0.485, 0.456, 0.406) and standard deviation (0.229, 0.224, 0.225). This tile extraction process is demonstrated in Fig 1, and breakdown of slides and sorted tiles can be found in Table 2.

**Table 2:** Model input data.

	Training		Testing	
	(n = 31)		(n = 16)	
Tissue samples (n, %)	213		117	
Samples per patient (mean, SD)	6.9 (2.3)		7.3 (1.9)	
Annotated Tiles (n, %)	ATARI	ResNet101	ATARI	ResNet101
Atrophy	3555 (38)	30000 (24)	1675 (38)	72098 (57)
G3	990 (11)	16000 (13)	475 (11)	14565 (11)
G4CG	130 (1)	5477 (4)	60 (1)	1078 (1)
G4NC	515 (6)	16482 (13)	235 (5)	5382 (4)
G5	75 (1)	4118 (3)	55 (1)	236 (<1)
HGPIN	285 (86)	4785 (4)	45 (1)	610 (<1)
Seminal Vesicles	435 (67)	10456 (8)	210 (5)	5728 (5)
Unlabeled Benign Tissue	3360 (67)	20000 (16)	1620 (37)	13483 (11)
Background	0 (0)	20000 (16)	0 (0)	14027 (11)
Total	9345	127319	4375 (32)	127207

Breakdown of annotated image tiles used for the training and testing datasets for the two models.

### Pathomic feature extraction

High resolution tiles were down-sampled to increase processing time, and then were processed using a custom, in-house MATLAB function to extract pathological features for use with the ATARI model. First, a color deconvolution algorithm was applied to each image to segment stroma, epithelium, and lumen based on their corresponding stain optical densities (i.e., positive hematoxylin or eosin, and background) [32]. These features were then further smoothed and filtered to remove excess noise and improve segmentations. Glands with lumen touching the edge of a tile were excluded. Overall stromal and epithelial areas were calculated on a whole-image basis, and an additional six features were calculated on an individual gland-basis: epithelial area, roundness, and wall thickness; luminal area and roundness, and cell fraction (i.e., the percent of epithelial cells per total gland area, defined by the area of the epithelium without lumen).

### Model training

Flowcharts for the ATARI model and ResNet101 classifier can be found in Fig 1. A bagged ensemble algorithm was used as the framework for developing the ATARI classifier on 31 subjects based in MATLAB (Mathworks, Inc. Natick, MA), which fitted predictors trained on bootstrapped samples from the training data set to obtain a combined ensemble model that minimized variance across learners [33, 34]. Bagged ensemble models prevent a single model from seeing the complete dataset and learns relationships across different components of the data, which reduces variance, prevents overfitting, and thus improves accuracy. Additionally, bagged ensembles are useful in cases where there are both linear and nonlinear data in the dataset, as would be assumed within histological data. Inputs for this model were mean, median, and variance of the calculated pathomic features averaged across each tile, z-scored across the training data.

To test a deep learning approach for comparison, a ResNet model with 101 layers was implemented in Python using the PyTorch framework (v.1.8.1) [20, 35]. The ResNet framework was chosen for the deep learning model due to its adaptable pre-trained framework, accuracy, and short training time. We found that the framework with 101 layers had the best performance in the model-tuning stage of our analysis. The same tiling procedure as previously described was used to curate the dataset for this network, with the addition of splitting all tiles into smaller 1024x1024 pixel patches and saving those that remained 50% within an annotation mask. Data from Cohort A was split into 80/20 training and validation datasets to prevent overfitting and several data augmentation techniques were used to increase training samples. The image patches were resized to 250x250 pixels, randomly cropped to 240x240 pixels, augmented and center cropped to generate the needed input size of 224x224 pixels. The same three model designs as the ATARI were trained using the ResNet101 framework. Class imbalance of the training dataset was addressed by introducing sample number-based class weights in the cross-entropy loss function. To test the granularity of Gleason pattern prediction, we trained predictive models using several different levels of tumor specificity including all Gleason grades; high- (G4+) and low-grade (G3) cancer and benign tissue (HG vs LG model); and non-cancer and cancer (G3+) (NC vs CA model). To test generalizability, the model was applied to a left-out test set. Predictions were then plotted on three slides from the test data set using the same features calculated across all tiles for the slide to assess successful identification of tumor and compared to ground-truth pathologist annotations and the ResNet model. All available code and generated models used for this study can be found at (https://github.com/sduenweg/Gleason_Annotation/tree/main) [36].

## Results

**Fig 2:**
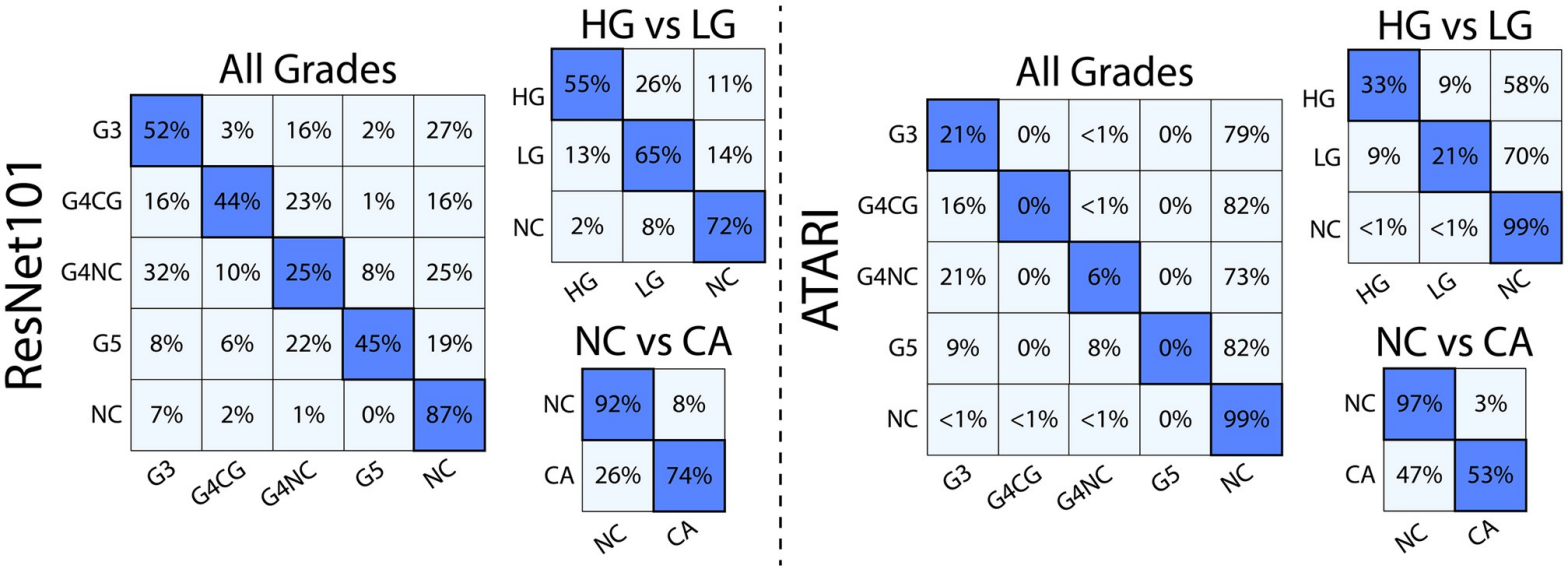
Model results. Confusion matrices for the three classification models for both the ResNet101 and ATARI. The ResNet101 was able to distinguish between unique Gleason patterns at higher accuracies that the corresponding ATARI models.

The accuracy of both models was analyzed using a left-out test dataset from 17 patients (95,875 image patches for the ResNet; 4,375 image tiles for ATARI). The ATARI model was unable to successfully classify Gleason grades (overall accuracy 85%, per-class accuracy range 0% - 99%) nor high- (HG) and low-grade (LG) cancer (overall accuracy 83%, per-class accuracy range <1% - 99%). In both models, normal tissue was classified well above chance level (20% for all Gleason grades, 33% for high- and low-grade cancer), with G3 in the Gleason grades model and HG in the HG vs LG model performing at chance. The ATARI non-cancer (NC) vs cancer (CA) model had an overall accuracy of 89% and a per class accuracy of 97% and 53% for NC and CA, respectively. The ResNet model was able to successfully classify all Gleason grades with an absolute overall accuracy of 79% (per class accuracy range 25% - 87%), HG vs LG (overall accuracy 72%, per class accuracy range 55% - 72%), and NC vs CA (overall accuracy 83%) with an accuracy 91% and 74% for non-cancer and cancer (Fig 2). The sensitivity of each annotation grade was typically higher in the ResNet models (0.45–0.8 for all Gleason grades, 0.74–0.9 for HG and LG) compared to ATARI (0–0.35 for all Gleason grades, 0.44–0.77 for HG and LG), except for G4NC (0.29 ResNet, 0.35 ATARI) and LG (0.66 ResNet, 0.68 ATARI). Similar results were observed for specificity and positive predictive value (PPV) (Table 3).

**Table 3:** Model performance.

		ResNet101				ATARI			
Model	Class	Sensitivity	Specificity	PPV	NPV	Sensitivity	Specificity	PPV	NPV
**All Grades**	G3	0.45	0.87	0.52	0.84	0.31	0.82	0.21	0.88
	G4CG	0.68	0.87	0.44	0.95	0.00	0.80	0.00	1.00
	G4NC	0.29	0.82	0.25	0.84	0.35	0.81	0.06	0.97
	G5	0.80	0.88	0.45	0.97	0.00	0.80	0.00	1.00
	NC	0.50	0.97	0.90	0.78	0.24	0.97	0.97	0.21
**HG vs LG**	HG	0.79	0.81	0.60	0.91	0.77	0.74	0.33	0.95
	LG	0.66	0.84	0.71	0.80	0.68	0.71	0.21	0.95
	NC	0.74	0.94	0.88	0.86	0.44	0.97	0.98	0.36
**NC vs CA**	CA	0.90	0.78	0.74	0.92	0.95	0.67	0.53	0.97
	NC	0.78	0.90	0.92	0.74	0.67	0.95	0.97	0.53

Performance metrics across annotated classes for each of the tested machine and deep learning models. Abbreviations: HG = high grade; LG = low grade; NC = noncancer; CA = cancer; PPV = positive predictive value; NPV = negative predictive value.

**Fig 3:**
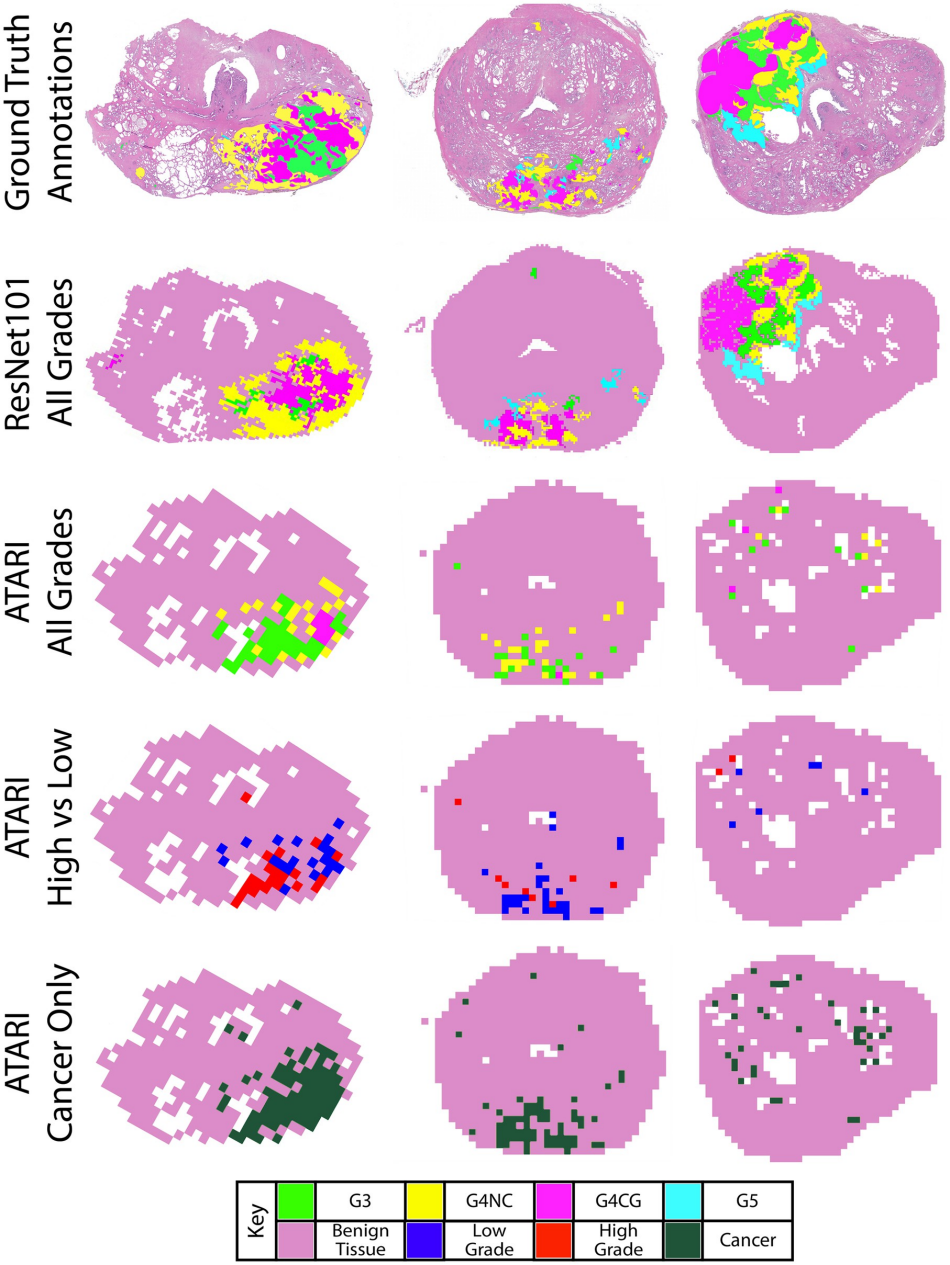
Example annotated slides. Ground truth annotation maps compared to the ResNet101 model for all Gleason grades and the three tested ATARI models: all Gleason grades, high- vs low-grade cancer, and cancer vs non-cancer only. ResNet101 model for all Gleason grades and the three ATARI models: all Gleason grades, high- vs low-grade cancer, and cancer vs non-cancer only.

Fig 3 show the representative slides as their ATARI and ResNet101 annotations as compared to ground-truth annotations. Although the ATARI model was unable to capture unique Gleason patterns, it was able to define the region of tumor present on the slide. The ResNet101 model was able to accurately predict the Gleason patterns with a per class accuracy of 25–52%.

## Discussion

In this study, high-resolution tiles taken from annotated regions on whole-mount digital slides after radical prostatectomy were used to train models to support pathologist diagnoses of prostate cancer. Specifically, the ATARI model used quantitative features to classify glandular features, whereas the ResNet101 classifier used deeper textural features of histology. The ATARI was only able to accurately predict cancer and non-cancer, whereas the ResNet101 classifier was able to further predict unique Gleason grades present on the slide. The results from our study indicate that while machine learning models using calculated features may be successful at differentiating tumor from non-tumor, deeper features found using neural networks can further define unique patterns. This may indicate that Gleason patterns exist beyond simple glandular features and may be more readably quantified using textural features. The absolute accuracies of 89% and 83% for the ATARI and ResNet101 models, respectively, show the need for a more general approach to using machine learning for cancer diagnosis.

The models tested in this study used divergent inputs per their algorithm requirements that may have resulted in their conflicting performances. The ATARI model was trained using eight quantitative pathomic features calculated across 3000x3000 pixel tiles as input (n = 9345), and the mean, median, and variance of each feature was calculated across tiles, resulting in 224,280 overall features. The ResNet model used the 1024x1024 pixel tiles themselves (n = 127319) and applied image augmentations to increase the number of examples in the training set and introduce more variety in what the model can learn from. This imbalance may have aided the ResNet in determining finer details to differentiate Gleason patterns, resulting in an overall accuracy, sensitivity, specificity, and positive predictive value across each annotation class for each model that generally was higher than those seen in the ATARI models. The ATARI model did, however, match or outperform the ResNet’s PPV in each model for the benign tiles, further indicating its ability to detect cancer from non-cancer.

Machine and deep learning applications are becoming prominent in clinical research. Machine learning focuses on the use of data and algorithms to imitate the way that humans learn. Data used in machine learning applications are human-derived, quantitative metrics that are then analyzed through statistical methods to make classifications or predictions. Deep learning is a sub-field of machine learning that automates the feature extraction without the need for human intervention. It can uncover more nuanced patterns within the data to generate predictions. In this study, our proposed machine-learning model outperformed the ResNet model at classifying cancer from non-cancer; however, the ResNet could classify unique Gleason grades. This may indicate that the features of Gleason grades do not have strong quantitative differences, but rather texture differences that are discernible using a deep learning model. This was especially true in the case of Gleason pattern 5, which the ATARI model had 0% accuracy in detecting. It would have been expected that the G5 pattern would be clearly discernible from the other Gleason patterns as it contains densely packed cells with small lumen, however, our pathomic feature calculator relies on the appearance of a distinct gland that has a clear epithelial wall around the lumen, which may be a limitation of the pathomic feature calculator compared to the deep learning. Additional pathomic features may increase the accuracy in detecting this pattern specifically. Other prior studies have shown similar results where a trained deep learning model outperformed a simple model trained on handcrafted features [37–39].

Automated Gleason grading applications have been previously applied for multiple purposes. One prior study trained a convolutional neural network (CNN) using WSI-level features constructed from a CNN-based PCa detection model that was trained from slide-level annotations to predict the final patient Gleason Grade Group [40]. This model achieved a 94.7% accuracy at detecting cancer and 77.5% accuracy at predicting the patient Grade Group. While promising, this model does not provide histological annotations to WSI, but rather only predict patient Grade Group. Several previous studies have applied deep learning models to prostate biopsy specimens [15, 41, 42]. While these models have achieved high accuracies at annotating biopsy cores, our ResNet101 model was able to annotate whole-slides images and could distinguish between regions of Gleason 4 cribriform and non-cribriform tumors. This distinction in detecting underlying features of G4CG and G4NC tumors is especially important to pathologists, as cribriform glands specifically have worse prognostic differences and thus requiring more aggressive treatment than the G4 non-cribriform patterns.

Integrating rapid annotation of Gleason patterns after tissue resection into the clinical workflow could save a tremendous amount of pathologist time. Once slides are digitally scanned, a diagnosis could be predicted automatically based on the automated annotations. This could then be used to rank slides by order of importance for pathologist review and to aid in treatment planning. The proposed models could be applied to large data sets and would decrease the workload on pathologists. Additionally, annotations provided from quantitative metrics may eliminate variability in Gleason annotations.

One major limitation of the study is the use of only one pathologist for annotating the training and test datasets. Inter-observer variability is a known issue in prostate cancer diagnosis, and thus should be addressed in the training phase. Additionally, only one slide scanner was used to digitize the slides used in this study. Future studies should investigate the impact additional slide scanners would have on the generalizability of the models, as this analysis was outside the scope of the current study. The models trained in this study also used input images of different sizes for training. While the ResNet model used a smaller input image with greater detail in small regions, the ATARI model used larger tiles for more accurate pathomic feature calculation across entire glands. Glands were often clipped in the smaller tiles used for the ResNet model in a way that would have made computed features unrepresentative of the actual histology, which limits the image resolution in a way that textural features used in deep learning models are not. Further research is warranted to determine the impact that input image sizes have on deep learning models. Finally, future studies should look at larger populations to provide a more robust dataset of Gleason patterns which may increase accuracy in the machine learning models, as this study had a relatively small cohort of 47 patients.

## Conclusion

We demonstrate in a cohort of 47 patients that machine learning models and neural networks can accurately predict regions of prostate cancer, where the latter network was further able to classify unique Gleason patterns. These models are anticipated to aid in prostate cancer decision support by decreasing the diagnostic burden of pathologists. Future studies should determine how inter-observer and slide scanner resolution impact these networks in their classifications.
